# Design and Development of a Novel Vaccine for Protection against Lyme Borreliosis

**DOI:** 10.1371/journal.pone.0113294

**Published:** 2014-11-19

**Authors:** Pär Comstedt, Markus Hanner, Wolfgang Schüler, Andreas Meinke, Urban Lundberg

**Affiliations:** Valneva Austria GmbH, Vienna, Austria; University of North Dakota School of Medicine and Health Sciences, United States of America

## Abstract

There is currently no Lyme borreliosis vaccine available for humans, although it has been shown that the disease can be prevented by immunization with an OspA-based vaccine (LYMErix). Outer surface protein A (OspA) is one of the dominant antigens expressed by the spirochetes when present in a tick. The *Borrelia* species causing Lyme borreliosis in Europe express different OspA serotypes on their surface, *B. burgdorferi* (serotype 1), *B. afzelii* (serotype 2), *B. garinii* (serotypes, 3, 5 and 6) and *B. bavariensis* (serotype 4), while only *B. burgdorferi* is present in the US. In order to target all these pathogenic *Borrelia* species, we have designed a multivalent OspA-based vaccine. The vaccine includes three proteins, each containing the C-terminal half of two OspA serotypes linked to form a heterodimer. In order to stabilize the C-terminal fragment and thus preserve important structural epitopes at physiological temperature, disulfide bonds were introduced. The immunogenicity was increased by introduction of a lipidation signal which ensures the addition of an N-terminal lipid moiety. Three immunizations with 3.0 µg adjuvanted vaccine protected mice from a challenge with spirochetes expressing either OspA serotype 1, 2 or 5. Mice were protected against both challenge with infected ticks and *in vitro* grown spirochetes. Immunological analyses (ELISA, surface binding and growth inhibition) indicated that the vaccine can provide protection against the majority of *Borrelia* species pathogenic for humans. This article presents the approach which allows for the generation of a hexavalent vaccine that can potentially protect against a broad range of globally distributed *Borrelia* species causing Lyme borreliosis.

## Introduction

Lyme borreliosis (LB) is an emerging disease and the most common vector-borne infection in the Northern hemisphere. The Center for Disease Control and Prevention (CDC) recently presented an updated estimate of 300,000 cases annually in the US [1]. This is almost a 10-fold increase to earlier estimations and would indicate that the disease is much more prevalent than previously thought. In Europe, it is only possible to provide approximate numbers of LB cases, since only few countries classify LB as a notifiable disease and underreporting is known to be pronounced. However, incidence based on notified cases and qualified estimates reports 65,000–85,000 cases annually in Europe [2]–[4].

Outer surface protein A (OspA) has been the basis for at least two different vaccines targeting LB; LYMErix (SmithKline Beecham) and ImuLyme (PasteurMérieux-Connaught). Both vaccines contained only OspA from *B. burgdorferi* as antigen, but only LYMErix was licensed and available for customers from 1998–2002, when it was voluntarily withdrawn from the market. OspA is a surface exposed lipoprotein of ∼28.5 kD [5], which is attached to the outer membrane by its N-terminal lipid moiety [6]. Thus, the C-terminal half is more distant from the bacterial surface and therefore more accessible for anti-OspA antibodies [7], [8]. OspA is abundantly expressed when spirochetes are located in the gut of the unfed tick and studies have shown that the passive transfer of OspA antibodies to mice protects them from infection when challenged with infected ticks [9]. OspA has been proposed to function as an antibody-shield in the tick during the blood meal from immune competent hosts [10]. Furthermore, the protein has been suggested to cover other conserved surface proteins such as P13 and P66 and thereby protect them from antibody recognition [11], [12]. Other functions assigned to OspA include adhesion activities mediating binding to plasminogen [13] and to TROSPA (tick receptor for OspA) on the gut epithelium [14]. During tick feeding OspA expression is down regulated, allowing the spirochetes to penetrate the gut epithelium, migrate to the salivary glands and further into the blood of the host [15]. Therefore, OspA-based LB vaccines act on spirochetes in the tick gut [9], where spirochetes are neutralized by anti-OspA antibodies in a complement independent manner, before they can infect the vertebrate host [16], [17].

Previous studies have shown that antibodies targeting the C-terminal part of OspA play a crucial role in protection [18]. Subsequently, it has been shown that a vaccine based on the C-terminal half of OspA from *B. burgdorferi* (constituting approximately 55% of the full-length protein) partially protected mice against a homologous challenge [19]. The introduction of mutations which facilitated hydrophobic interactions increased the stability and protection of the C-terminal half of OspA to levels comparable to full-length OspA. This demonstrated the necessity not only to include regions important for protection in a vaccine, but also to ensure that the structure is maintained to induce a protective immune response.

In Europe, four *Borrelia* species representing six OspA serotypes (*B. burgdorferi* (serotype 1), *B. afzelii* (serotype 2), *B. garinii* (serotype, 3, 5 and 6) and *B. bavariensis* (serotype 4)) cause the majority of infections in human, whereas in North America, only *B. burgdorferi* (serotype 1) is found [20]–[24]. In addition, *B. spielmanii, B. lusitaniae, B. bissettii* and *B. valaisiana* have been isolated from patients diagnosed with LB, showing that the disease can be caused also by less common *Borrelia* species [25]–[30]. The development of a vaccine effectively targeting all these *Borrelia* species expressing different OspA serotypes is challenging, because the vaccine has to induce protective immunity against each individual OspA serotype and at the same time the number of proteins in the final formulation should be kept low. The Valneva approach addresses these challenges by (1) using only the C-terminal domain of the six most clinically relevant OspA serotypes, (2) introducing mutations which facilitate the formation of stabilizing disulfide bonds to retain the thermal stability needed for protection and (3) linking the six truncated OspA proteins (monomers) together to form three heterodimers each expressed with a lipid moiety. In addition, the putative T cell epitope in OspA from *B. burgdorferi* presenting homology to human leukocyte function-associated antigen-1 (hLFA-1) and claimed to induce antibiotic-refractory Lyme arthritis in a subset of naturally infected patients [31], has been eliminated through the replacement by corresponding sequence from *B. afzelii*. This precaution has been taken even though the immune response induced by an OspA based vaccine has been shown to not replicate the events needed to induce Lyme arthritis in a natural infection [32]. The post-translational addition of a lipid moiety is a critical determinant for the immunogenicity of OspA [33]. The vaccine including the three heterodimers formulated with aluminium hydroxide (the LB-vaccine), was tested for protection in mouse models where both ticks infected with *B. afzelii* (serotype 2) and *in vitro* grown *B. burgdorferi* (serotype 1) and *B. garinii* (serotype 5) were used for challenge. The induction of a broad immune response was further analyzed using spirochetes expressing the six clinically most relevant OspA serotypes. Binding of LB-vaccine induced antibodies to “native” epitopes of OspA presented on the surface of spirochetes and functionality of the antibodies were analyzed by flow cytometry and growth inhibition assays, respectively. This article presents the design and efficacy of a novel, next generation OspA-based LB-vaccine with the capacity for broad protection.

## Materials and Methods

### Ethics statement

All animal experiments were conducted in accordance with Austrian law (BGBl No. 114/2012) and approved by “Magistratsabteilung 58″. Experimental procedures were reviewed and approved by the Valneva animal welfare committee and experiments performed by specially trained personnel. Animals were anaesthetized by an injection of ketamin and xylazine when the ventilated containers were mounted. Isoflurane was used when ticks were applied to the mice and for the collection of terminal blood. Every effort was made to minimize suffering.

### Protein modeling

The crystal structures of OspA from *B. burgdorferi* (serotype 1) when bound to monoclonal antibodies 184.1 (N-terminal epitope) or LA-2 (C-terminal epitope) and the structure of a modified OspA were used as basis for the 3D-modeling of the C-terminal OspA fragment [7], [34], [35]. The structures were modeled using the open-source version of PyMOL (http://sourceforge.net/projects/pymol/) [36]. Sites geometrically suitable for the formation of disulfide bonds were identified by manual inspection and by using DSDBASE, which applies the computational procedure MODIP (modeling of disulfide bonds in proteins) [37]. The corresponding amino acid positions in OspA from *B. afzelii* (serotype 2) were substituted with cysteines to enable the formation of disulfide bonds. The finally selected position for stabilization (modification “B”) was also translated to the analogous positions of OspA serotypes 1, 3–6.

### Cloning

The constructs used for expressing the *B. afzelii* OspA monomers were derived from the amino acid sequence of strain K78 and were codon optimized for *E. coli* expression (GenScript, USA). The *ospA* nucleotide sequence encoding the monomer proteins (aa 131–273) were inserted into pET28b(+) (Merck Millipore, USA) using the restriction enzymes *Nco*I and *Xho*I, while the fragments encoding the lipidated (abbreviated “Lip”) monomers (aa 128–273) were inserted using *BspH*I and *Xho*I. The OspA monomers (abbreviated “M” followed by the corresponding OspA serotype and a letter (A to E) indicating the stabilization), were expressed in BL21 Star (DE3) (Invitrogen, USA). The expression plasmids for the heterodimers (abbreviated “D” followed by the corresponding OspA serotype and a letter indicating the stabilization of the first and second monomer) were generated by first constructing a vector containing 23 aa including the signal sequence (MKATKLVLGAVILGSTLLAGCSS) from the *E. coli* major outer membrane lipoprotein (Lpp). In each case the first OspA monomer inserted between the lipidation signal and the linker (*Hind*III–*Spe*I) of the three expression plasmids was either the C-terminal OspA fragment from *B. burgdorferi* serotype 1 (aa 126–273, strain B31, GenomeNet accession number NP_045688.1), *B. bavariensis* serotype 4 (aa 126–273, strain PBi, NCBI accession number YP_063283.1) or *B. garinii* serotype 5 (aa 126–273, strain PHei, EMBL accession number CAA56544.1). The middle fragment (*Spe*I–*Sca*I; linker) is the combined sequence from two separate loop-regions (aa 65–74 and aa 43–53, with an amino acid exchange at position 53 [D53S]) of OspA serotype 1 (strain B31). The second OspA monomer inserted respectively after the linker (*Sca*I–*Xho*I) was either the C-terminal OspA fragment from *B. afzelii* serotype 2 (aa 126–273, strain K78, Valneva Austria GmbH sequence data), *B. garinii* serotype 3 (aa 126–274, strain PBr, NCBI accession number YP_002476925.1) or *B. garinii* serotype 6 (aa 126–274, strain DK29, EMBL accession number CAA45010.1) and has a stop codon prior to the *Xho*I restriction site. All OspA fragments of the heterodimers were stabilized with a disulfide bond (stabilization “A” or “B”). In addition, the hypothetical molecular mimicry between a T cell epitope of OspA (serotype 1) and hLFA-1 (human leukocyte function-associated antigen-1) was eliminated by replacing the sequence of *B. burgdorferi* OspA with the corresponding region from *B. afzelii* OspA.

### Protein expression and purification

Expression of all monomers in BL21 Star (DE3) cells was induced with 0.1 mM IPTG and the temperature was lowered to 25°C during induction. After overnight induction, cells were pelleted by centrifugation and disrupted mechanically by high pressure homogenization. Lipidated monomers were purified by phase separation with Triton X-114 prior to affinity purification. The soluble protein fractions were applied to a Ni-Sepharose 6 Fast Flow column (GE Healthcare, United Kingdom) and the bound His-tagged proteins were eluted with an Imidazole gradient (0–250 mM), followed by a buffer exchange column (Sephadex G-25, GE Healthcare). Expression of the lipidated heterodimers was induced at 37°C for 3 to 4 h with 1 mM IPTG. Proteins were extracted by phase separation with Triton X-114 and purified with a combination of Q-Sepharose (GE healthcare), hydroxyapatite (Bio-Rad, USA) and DEAE-Sepharose (GE healthcare) chromatographic steps. Finally the buffer was changed using a Superdex 200 gel filtration column (GE healthcare). Samples from different purification steps were separated on 15% SDS-PAG and stained with Coomassie blue (Sigma-Aldrich, USA). The protein concentrations were determined using Pierce BCA protein assay kit (Thermo-Scientific, USA). After sterile filtration, all purified recombinant proteins were stored at −80°C.

### Thermal stability

The fluorescent dye SYPRO orange protein gel stain (Sigma-Aldrich, USA) was used to monitor protein denaturation as described previously [38]. To each well, 7.5 µL of SYPRO Orange (1∶1,000 diluted) and 17.5 µL protein (2 µg) was added. The proteins were analyzed with the CFX96 Real-time Detection System (Bio-Rad) and changes in fluorescent intensity were monitored (excitation at 490 nm and emission at 575 nm) during heating. The temperature was increased from 25 to 95°C at a rate of 0.2°C/10 s. Calculations of T_m_ were performed with the Bio-Rad CFX Manager 2.0 program. The T_m_ determinations were performed in a buffer consisting of 50 mM Tris-HCl and 150 mM NaCl (pH 8.0).

### Immunization of mice

Eight-weeks-old female C3H/HeN mice were used for all studies (Janvier, France). Mice were immunized subcutaneously (s.c.) three times with 100 µL vaccine at two-week intervals. All vaccines were formulated with 0.15% aluminum hydroxide, if not otherwise stated. One week after the third immunization, blood was collected and hyper-immune sera were prepared.

### Challenge with *B. afzelii* infected ticks

The *B. afzelii* strain IS1, a field isolate never passaged *in vitro*, was used to infect *Ixodes ricinus* ticks by breeding them on infected gerbils (Insect Services, Germany) [39]. The infection prevalence of the ticks was >90%, as assessed by qPCR targeting the *recA* gene. Two weeks after the third immunization, the hair on the back of mice was removed with Veet Cream (Reckitt Benckiser, United Kingdom) and a small ventilated container was glued to the skin with super glue (Pattex, Germany). One day later, two infected *I. ricinus* nymphs were placed in the ventilated container and were allowed to feed until repletion. The feeding status of the ticks was monitored and only mice where at least one fully fed tick was collected were included in the final readout.

### Challenge with *in vitro* grown spirochetes

Two weeks after the last immunization, mice were challenged s.c. with 5×10^4^ *B. burgdorferi* strain ZS7 (OspA serotype 1) or 5×10^3^ *B. garinii* strain PHei (OspA serotype 5), diluted in 100 µL BSK-II medium [40]. The challenge doses correspond to between 5× and 10× ID_50_. Prior to each challenge, OspA expression was verified by flow cytometry as described below. Challenge of mice was only performed with cultures where >80% of cells were positive for OspA expression.

### Sacrifice of mice and collection of material

Four to six weeks after the challenge mice were sacrificed by cervical dislocation. Blood was collected by orbital bleeding and sera were prepared and used for VlsE ELISA to determine the infection status. In addition, one ear or the urinary bladder from each mouse was collected, DNA extracted and subjected to nested PCR or qPCR for identification of spirochetes.

### ELISA with the Invariable Region 6 (IR6) of VlsE

A biotinylated 25-mer peptide (MKKDDQIAAAMVLRGMAKDGQFALK) derived from the sequence of *B. garinii* strain IP90 was used for the analysis [41]. Streptavidin pre-coated 96-well ELISA plates (Nunc, Denmark) were coated with 0.1 µg peptide in PBS supplemented with 0.1% Tween-20 (PBS/0.1T) and incubated overnight at 4°C. Post challenge sera were tested at 1∶200 and 1∶400 dilutions in duplicates and incubated for 90 min at room temperature (RT). Polyclonal rabbit anti-mouse IgG conjugated to HRP (Dako, Denmark) was used as secondary antibody. ABTS was added as substrate (Sigma-Aldrich) and absorbance was measured at 405 nm. Negative controls included PBS instead of sera as well as plates not coated with the peptide. Sera from mice infected with *B. afzelii* were used as positive controls.

### Detection of spirochete DNA with PCR

The ear or urinary bladder from each mouse was subjected to DNA extraction using the DNeasy Blood and Tissue Kit (Qiagen, Germany) according to the manufacturer’s instructions with the following modifications. The tissue was incubated in 100 µL recombinant Proteinase K (PCR grade; Roche, USA) over night at 60°C. DNA was eluted in 50 µL deionized sterile water and stored at −20°C.

Nested PCR targeting the 16S–23S intergenic spacer was applied to detect spirochete DNA for assessment of the infection status of mice as described by Bunikis *et al*. [11]. Briefly, 1 µL from each DNA extraction was used for the first PCR reaction and 1 µL of the first reaction was used as sample for the second PCR reaction. Five microliter of the final reaction was analyzed on a 1% agarose gel. DNA purified from an *in vitro* grown culture of *B. afzelii* strain K78 was used as a positive control.

For protection experiments with the OspA heterodimers, qPCR was used for detection of spirochete DNA. Oligonucleotides for the *recA* gene were designed for identification of all relevant *Borrelia* species causing LB (forward: 5-CATGCTCTTGATCCTGTTTA-3, reverse: 5-CCCATTTCTCCATCTATCTC-3). The *recA* fragment was cloned from *B. burgdorferi* strain N40 into pET28b(+), to be used as standard in each reaction. DNA extracts were diluted in water to reduce the matrix effects and 2 µL was used for amplification using a CFX96 real-time PCR detection system (Bio-Rad); 50 cycles at 95°C for 15 s and 55°C for 30 s followed by preparation for melting curve analysis at 95°C for 30 s and 55°C for 2 min.

### Data analysis and infection readout

Infection readout was based on two different analyses; detecting the presence of spirochete DNA (nested PCR or qPCR) and spirochete specific antibodies (VlsE ELISA). There was a high consistency between the two methods (>95%); therefore a mouse was regarded as infected when at least one of the two methods was positive. Statistical significance was determined by Fisher's exact test (two-tailed).

### OspA ELISA

ELISA plates (Maxisorp, Nunc) were coated with 50 ng OspA protein in PBS and incubated at 4°C overnight. The plates were blocked with 1% BSA, 0.5% Tween-20, PBS for 1–2 h at RT. Plates were washed with PBS/0.1T. Individual sera from 10 mice were diluted in blocking buffer (five-fold dilution) and tested in duplicates by incubating for 1 h at RT. Plates were then washed with PBS/0.1T. The secondary antibody (horseradish peroxide [HRP] conjugated rabbit anti-mouse IgG, DAKO) was diluted 1∶2,000 in blocking buffer and incubated for 1 h at RT. Plates were washed with PBS/0.1T and ABTS (Sigma-Aldrich) was used as substrate, the reaction was stopped by the addition of 1% SDS and the absorbance was read at 405 nm. The half-max titer (the reciprocal of the dilution that corresponds to the mean absorbance between highest and lowest dilution) was determined.

### Flow cytometry

Spirochetes (1×10^6^) were fixed by the addition of an equal volume of 4% paraformaldehyde and incubation for 1–2 h at RT. Subsequently, cells were washed with HBSS +2% BSA (HBSS-B). Heat-inactivated sera from 10 mice were pooled, diluted in HBSS-B and sterile filtered using Costar spin-X centrifuge tube filters (0.22 µm, Corning, USA). Washed spirochetes were resuspended in 100 µL serum and incubated for 45 min at RT. The cells were washed with HBSS-B and subsequently resuspended in 100 µL HBSS-B. One microliter secondary antibody (PE conjugated goat anti-mouse IgG; Beckman Coulter, USA), was added and incubated for 45 min at RT in the dark. Spirochetes were washed with HBSS-B and then resuspended in 200 µL HBSS containing 5 µg LDS 751 (Life technologies, USA) and incubated for 10 min at RT in the dark. The stained spirochetes were pelleted by centrifugation and resuspended in 200 µL HBSS. Labeled spirochetes were measured with a FC500 (Beckman Coulter) flow cytometer, gated for LDS 751 positive events. Values obtained with sera from the placebo-immunized mice were subtracted from the values obtained with sera from mice immunized with the vaccines to compensate for non-specific binding. The surface binding data are presented as fluorescence titer, which is the reciprocal of the serum dilution corresponding to a fluorescence intensity of 100.

### Growth inhibition assay

Serial dilutions (1∶5) of heat-inactivated immune sera were incubated with spirochetes (2×10^3^–1×10^4^ cells) in the presence of 1% guinea pig complement (Sigma-Aldrich) for three to four days at 35°C and 1% CO_2_. The number of spirochetes was determined by flow cytometry after staining the cells with LDS 751. Sera from mice immunized with placebo as well as guinea pig complement alone were included as controls. All results are presented as growth inhibition titers, which is the reciprocal of the lowest serum dilution which gives ≥50% reduction in growth as compared to sera from mice immunized with placebo.

## Results

### Newly introduced disulfide bonds increase thermal stability of modified OspA monomers

It has previously been shown that antibodies binding to epitopes localized in the C-terminal half of OspA have been associated with protection [9], [42]. The importance of preserving structural epitopes of the C-terminal half of the protein when used for vaccine studies has also been demonstrated [19]. Therefore, we focused our approach for an OspA-based vaccine on the C-terminal half that includes the most relevant epitopes needed to induce protective immunity. To preserve structural epitopes of the C-terminal OspA fragment at physiological temperature, mutations resulting in a single stabilizing disulfide bond were introduced.

**Figure 1:**
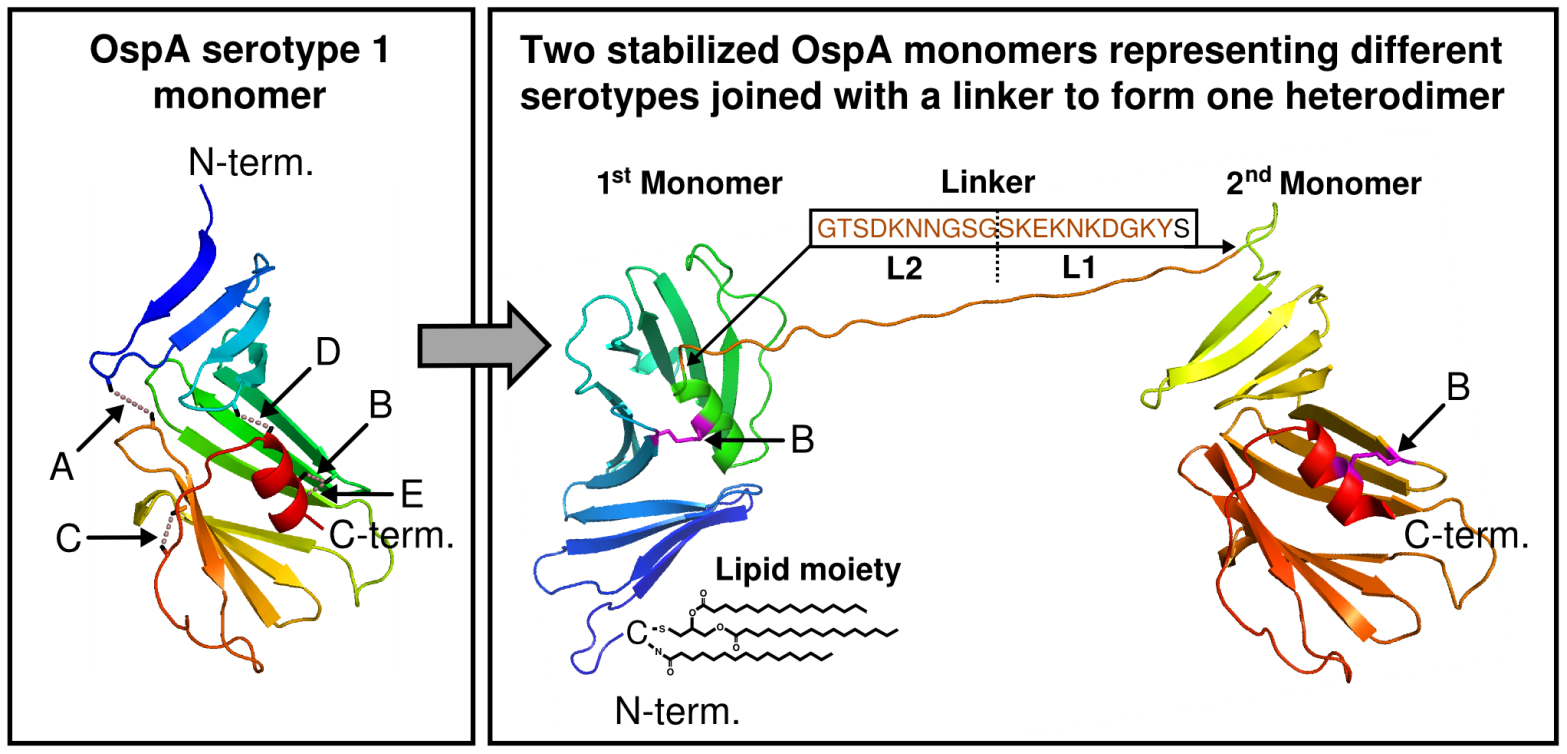
Localization of the stabilizing disulfide bonds and schematic representation of the heterodimers. Left panel: Part of the OspA serotype 1 crystal structure showing the monomer protein (Protein Data Bank accession 1OSP [34]). Locations of the five modifications: “A” (aa 141∶ 241), “B” (aa 182∶ 269), “C” (aa 244∶ 259), “D” (aa 165∶ 265) and “E” (aa 182∶ 272) are indicated as dotted lines between the β-carbon atoms of the corresponding residues. Right panel: Schematic illustration of two stabilized monomers joined with a linker sequence to form one heterodimer. The α-helix at the C-terminal end of the first monomer is joined with the β-sheet at the N-terminal domain of the second monomer using a linker sequence derived from *B. burgdorferi* OspA serotype 1 (L1; aa 43–53 with modification [D53S] and L2; aa 65–74). The heterodimer is expressed with a posttranslationally attached N-terminal lipid moiety, indicated in line drawing. The positions of the disulfide bonds for stabilization “B” in both OspA monomer subunits are indicated in magenta. Abbreviations: N-terminal (N-term.), C-terminal (C-term.).

Approximately fifty sites geometrically suitable for the formation of disulfide bonds based on their distance and orientation, as determined from three crystal structures [7], [34], [35], were identified in the C-terminal part of OspA from *B. burgdorferi*. Out of these, five sites were selected for their capability to bridge potentially weak regions of the structure, mainly between the β-sheets or α-helix secondary structure elements. The refined disulfide bond modeling procedure by Dani and co-workers [43], led us to set a cut-off of 4.7 Å for the distance between β-carbon atoms (being the anchor positions for sulfur in a cysteine) of the selected sites. The positions of the selected amino acids (aa) for the different mutations (A to E) and their β*-*carbon atom distances are specified in Table 1 and graphically presented in Figure 1, left panel. The newly formed disulfide bond resulting from modification “A” exceeds the cut-off value and therefore likely brings the involved amino acids closer together as compared to the native structure. However, both mutated residues are located in loop-regions which allow for some local flexibility upon disulfide-bond formation without necessarily affecting the overall fold of the protein.

**Table 1:** Selected positions for the introduction of cysteines and calculated distances between the respective β-carbon atoms.

	Amino acid positions^a^		β-carbon atom distances^b^		
Modification	1^st^ aa	2^nd^ aa	mAb 184.1	mAb LA-2	modified OspA
A	141 (Asp)	241 (Asn)	6.0 Å	6.2 Å	6.1 Å
B	182 (Glu)	269 (Lys)	4.2 Å	4.1 Å	3.9 Å
C	244 (Thr)	259 (Ala)	4.0 Å	4.1 Å	4.1 Å
D	165 (Tyr)	265 (Leu)	4.0 Å	4.2 Å	4.3 Å
E	182 (Glu)	272 (Leu)	3.0 Å	3.9 Å	4.3 Å

a Positions and residue indicated in parentheses for the 1^st^ and 2^nd^ amino acid (aa) corresponding to the five selected modifications (A to E). ^b^ Distances in Ångström (Å) between the two β-carbon atoms based on the crystal structures of full-length OspA from *B. burgdorferi* (serotype 1) bound to mAb 184.1 (N-terminal epitope), mAb LA-2 (C-terminal epitope) and a modified OspA [7], [34], [35].

We used the C-terminal OspA fragment (aa 131–273) referred to as monomer (abbreviated “M”), from *B. afzelii* OspA serotype 2 for our proof-of-concept studies to evaluate five different mutations for stabilization with regards to increased thermal stability and vaccine efficacy. The amino acids at the analogous positions in OspA serotype 2 were therefore substituted with cysteines to enable the formation of disulfide bonds. Consequently, five His-tagged OspA serotype 2 monomers were produced; M2A-His, M2B-His, M2C-His, M2D-His and M2E-His (the mutation for each monomer is indicated with the letter A to E and their locations are outlined in Table 1). All five mutations introduced for the formation of disulfide bonds, resulted in stabilization of the monomers as determined by experiments assessing their thermal stability under non-reducing and reducing (in the presence of DTT) conditions (Table 2). Four of the mutated monomers (M2A-His, M2B-His, M2C-His and M2E-His) showed an increased melting temperature (T_m_) under non-reducing conditions as compared to reducing conditions, indicating the formation of a disulfide bond. The monomer M2D-His showed similar T_m_ for both conditions and the formation of a disulfide bond could therefore not be verified with this method. However, all of the modifications expect M2C-His showed even an increased T_m_ under reducing conditions as compared to the unmodified truncated protein, M2-His (Table 2).

**Table 2:** Introduction of a disulfide bond increases the thermal stability of OspA monomers.

	Melting temperatures^a^	
OspA monomer	Non-reducing	Reducing
M2-His	47.6°C	47.0°C
M2A-His	58.4°C	54.8°C
M2B-His	70.4°C	58.4°C
M2C-His	58.4°C	45.2°C
M2D-His	54.5°C	54.9°C
M2E-His	55.3°C	52.2°C

a T_m_ in degrees Celsius (°C) under non-reducing (without DTT) and reducing (with DTT) conditions.

### Immunization with stabilized OspA monomers protects mice from infection transmitted by *B. afzelii* infected ticks

The six OspA monomers were tested in a mouse model for protection against challenge with *B. afzelii* infected ticks. All proteins were initially expressed without a lipidation signal, but with a C-terminal His-tag (M2-His, M2A-His, M2B-His, M2C-His, M2D-His and M2E-His) to facilitate purification. Mice were immunized subcutaneously (s.c.) three times with 5.0 µg OspA monomer formulated with 0.15% aluminum hydroxide at two-week intervals. Due to the complexity of the tick challenge model, the six different proteins were evaluated in separate experiments following an identical protocol, using the same batch of infected ticks and performed by the same technical staff. Consequently, the data presented in Table 3, summarize the results from four experiments, where only mice with at least one fully fed tick were included in the evaluation. The high infection rates of the placebo groups together with the high level of protection generated by the positive control, OspA2-His (full-length OspA serotype 2 without a lipid moiety) validated the experiments. All five stabilized OspA monomers generated highly significant levels of protection when mice were challenged with ticks harboring *B. afzelii* (serotype 2). However, immunizations with the unmodified truncated OspA (monomer M2-His) provided only partial protection which was not significant when compared to the placebo group (Table 3). Since all five OspA monomers provided high levels of protection in the mouse tick challenge model, selection of stabilizing mutations for further studies was based on the level of protein expression and phase separation. When expressed in *E. coli*, the three monomers with the stabilizations A, B or C resulted in more biomass per liter growth medium as compared to the other two monomers (D and E). The monomer M2B-His had the highest recovery after purification (Table 4). Cells expressing M2D-His did not reach high densities nor resulted in considerable amounts of purified protein. Even though M2E-His showed overall acceptable expression levels, the high similarity to M2B-His (modification of residues 182 and 272 or 182 and 269, respectively) led us to discontinue this candidate. Taking all protein expression and purification yields into consideration, we therefore selected modifications A, B and C for our further studies.

**Table 3:** Stabilized OspA monomers are protective in a mouse model using *B. afzelii* infected ticks for challenge.

Treatment		Infected/Total^a^	
Immunogen	Dose	Vaccine	Placebo
OspA2-His	5.0 µg	1/25***	20/23
M2-His	5.0 µg	8/16^n.s.^	13/15
M2A-His	5.0 µg	4/27***	20/23
M2B-His	5.0 µg	1/25***	20/23
M2C-His	5.0 µg	1/21***	20/23
M2D-His	5.0 µg	2/11**	7/8
M2E-His	5.0 µg	4/26***	20/23

Immunized mice were challenged with ticks harboring *B. afzelii* and the infectious status was determined six weeks later by screening for the presence of spirochete specific antibodies (VlsE ELISA) and DNA (nested PCR targeting the 16S–23S intergenic spacer), using terminal sera and ear tissue respectively. Mice were regarded as infected when at least one of the two methods was positive.

a Data from four experiments following the same protocol have been combined and only data from mice where at least one fully fed tick was collected are presented. The *p-*values were calculated with Fisher’s exact test (two tailed); ** *p*<0.01, *** *p*<0.001 and ^n.s.^ not significant.

**Table 4:** Biomass and yield of purified OspA monomers.

OspA monomer	Biomass/Liter (g)	Protein/gram biomass(mg/g)
M2A-His	17.1	1.3
M2B-His	10.3	16.4
M2C-His	14.0	2.5
M2D-His	6.4	1.3
M2E-His	6.6	7.0

The five different OspA monomers were evaluated with regards to *E. coli* growth (biomass/Liter) and the amount of protein purified.

In order to improve immunogenicity even further, a signal peptide for lipidation was inserted to produce three new lipidated OspA serotype 2 monomers (Lip-M2A-His, Lip-M2B-His and Lip-M2C-His). Protection was assessed using the mouse tick challenge model as described above. As an attempt to further distinguish the three proteins based on their protective capacity, mice were immunized with two doses (3.0 µg or 0.3 µg) of protein formulated with 0.15% aluminum hydroxide. Data from three and six individual experiments, respectively, were combined and are presented in Table 5. Reproducibly, the placebo groups showed high infection rates whereas the positive control groups, mice immunized with full-length OspA from *B. afzelii* (OspA2-His) were highly protected. The un-lipidated positive control was at this stage only used to validate the mouse tick challenge model and not as a bench mark for protection of the monomers. All three stabilized OspA monomers expressed with an N-terminal lipid moiety provided highly significant levels of protection at both doses tested when compared to the respective placebo group. However, a small reduction in protection could be observed at the lower immunization dose for mice having received the OspA monomer Lip-M2C-His.

**Table 5:** Lipidated, stabilized OspA monomers induce protection in mice against challenge with *B. afzelii* infected ticks.

Treatment		Infected/Total^a^	
Immunogen	Dose	Vaccine	Placebo
OspA2-His	3.0 µg	0/14***	23/24
	0.3 µg	1/17***	15/15
Lip-M2A-His	3.0 µg	0/8***	10/10
	0.3 µg	0/34***	34/34
Lip-M2B-His	3.0 µg	0/17***	13/14
	0.3 µg	1/31***	35/35
Lip-M2C-His	3.0 µg	1/15***	13/14
	0.3 µg	5/19***	20/20

a Data from three and six experiments (3.0 µg and 0.3 µg immunization dose group, respectively) following the same protocol have been combined and only data from mice where at least one fully fed tick was collected are presented. The *p-*values were calculated with Fisher’s exact test (two tailed); *** *p*<0.001.

### Production of heterodimers by fusion of stabilized OspA monomers representing two serotypes is facilitated by the “B”-type stabilization

**Figure 2:**
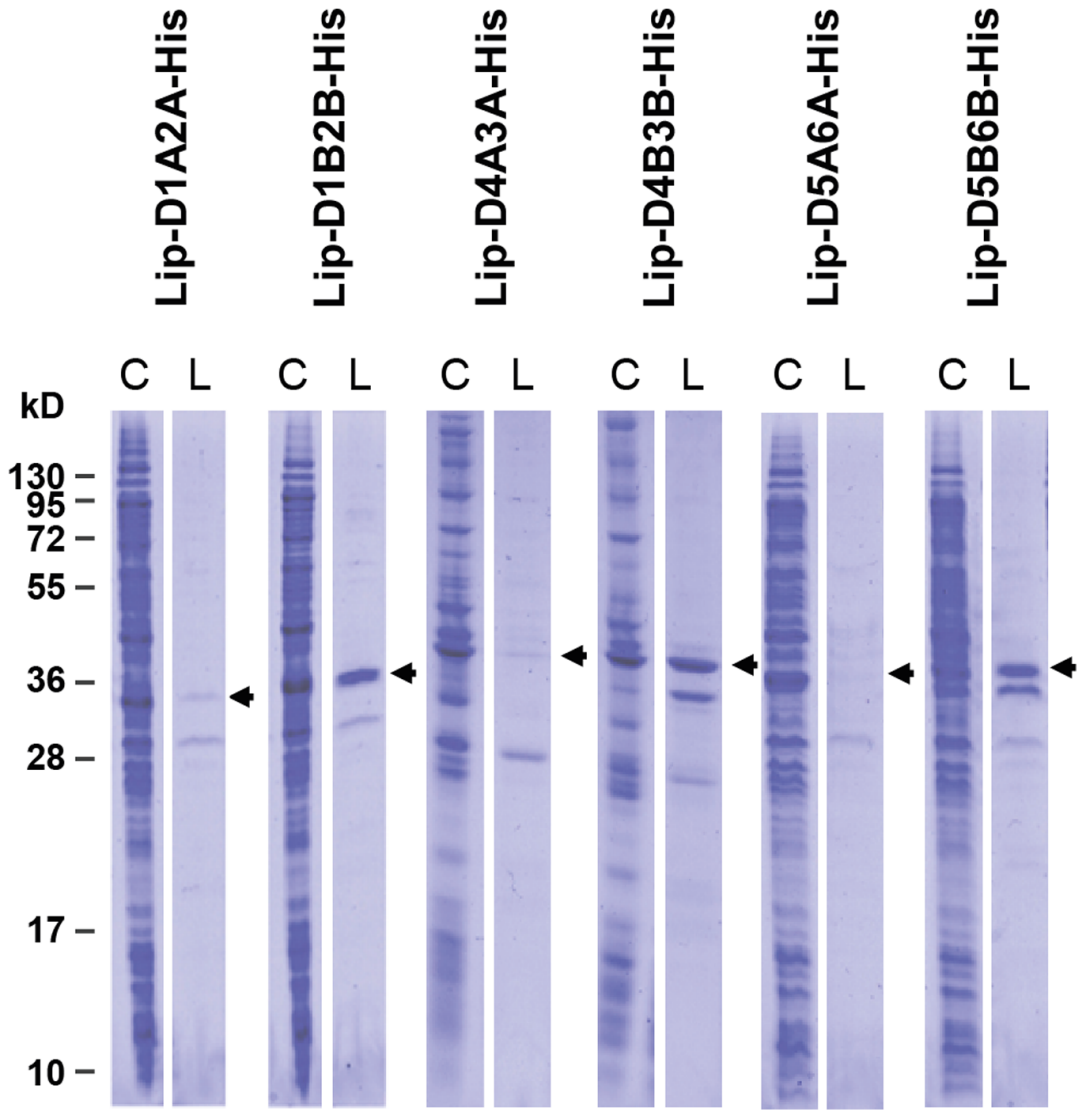
Protein expression and lipid-phase separation for OspA heterodimers. Each heterodimer was expressed in two different versions having the respective monomers stabilized with the “A” or the “B” modifications; Lip-D1A2A-His, Lip-D1B2B-His, Lip-D4A3A-His, Lip-D4B3B-His, Lip-D5A6A-His and Lip-D5B6B-His. The expression levels of all six heterodimers were similar when the crude lysates were analyzed. However, heterodimers using stabilization “B” were recovered in higher amounts in the lipid-phase after phase separation, indicating more efficient lipidation and higher solubility. Protein bands representing the heterodimers are indicated with arrows. Abbreviations: Crude lysate (C), Lipid-phase (L).

In order to target the majority of LB causing *Borrelia* species, the stabilizing modifications had also to be translated to OspA serotype 1, 3–6. The C-terminal part of OspA from *B. afzelii* (aa 126–273), has a sequence identity of 68–86% to the other five OspA serotypes. This is in a range where structural fold conservation can be expected which was further supported by analyzing homology models of the five OspA serotypes based on the crystal structure of OspA serotype 1 [44]. The differences in distance between the β-carbon atoms, where the cysteines are introduced, were 0.0–0.2 Å when the five modifications in serotype 2 to 6 were compared to the two crystal structures of OspA serotype 1 (co-crystallized with mAb 184.1 or mAb LA-2). Therefore, the transfer of the position for substitutions to other OspA serotypes could be performed in a straightforward manner. The region in OspA serotype 1 having similarities to hLFA-1 was eliminated by replacement with the homologous sequence from OspA serotype 2. Since modifications “A” and “B” were comparable based on thermal stability and protection data, both were studied further. However, to reduce the number of proteins in the final vaccine, OspA monomers representing two different serotypes were linked at this point to form heterodimers (Figure 1, right panel). Monomers from OspA serotype 1 and 2, serotype 4 and 3, and serotype 5 and 6 were therefore joined with a linker. A 21 amino acid sequence derived from two loops of the N-terminal half of OspA from *B. burgdorferi* were used as linker. Thus, two different variants of each heterodimer were generated, where each monomer pair contained either the “A” or “B” modification. Even though the expression of all six heterodimers were similar, the three variants using stabilization “B” showed a higher recovery after phase separation, which indicated more efficient lipidation and higher solubility (Figure 2). Consequently heterodimers (abbreviated “D” followed by a number for the OspA serotype and a letter for the cysteine modification of the first and second monomer subunit respectively as presented in Table 1); Lip-D1B2B, Lip-D4B3B and Lip-D5B6B were selected for evaluation of vaccine efficacy for single heterodimers and for the combination of all three heterodimers (referred to as LB-vaccine).

### The LB-vaccine induces a serotype specific immune response and protects mice against both tick and subcutaneous challenge

**Figure 3:**
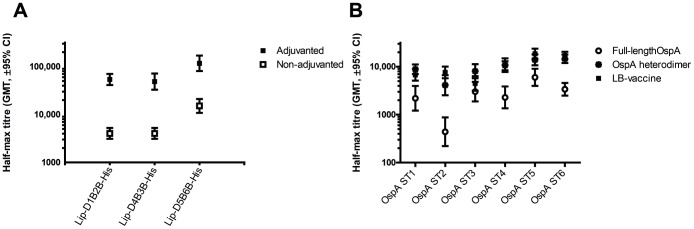
Immunogenicity of OspA heterodimers. (A) The effect of adjuvant on the immune response was evaluated. The three heterodimers (Lip-D1B2B-His, Lip-D4B3B-His and Lip-D5B6B-His) were tested individually for immunogenicity (5.0 µg) when formulated with or without adjuvant (0.15% aluminum hydroxide). IgG titers were determined by ELISA using the respective heterodimer as coating antigen. The half-max geometric mean titers (GMT) with 95% confidence intervals (CI) are shown. (B) The serotype specific immune response of the LB-vaccine was studied by immunizing mice with 3.0 µg LB-vaccine (Lip-D1B2B, Lip-D4B3B and Lip-D5B6B in a 1∶1∶1 ratio), or 1.0 µg of the heterodimers or the homologous lipidated full-length OspA, with adjuvant. IgG titers were determined by ELISA using the monomer of the corresponding serotype (ST1–6) as coating antigen. The half-max geometric mean titers (GMT) with 95% CI are shown.

The mode of action of an OspA based vaccine is to induce antibodies that eliminate the spirochetes in the gut of the feeding tick and thereby block the transmission to the vertebrate host [9]. This requires high titers of circulating antibodies and a strong humoral immune response is therefore needed. For that reason we studied the effect of an adjuvant primarily stimulating the humoral immune response. Mice were immunized s.c. three times with 5.0 µg of the individual heterodimers (Lip-D1B2B-His, Lip-D4B3B-His and Lip-D5B6B-His) formulated with or without aluminum hydroxide. Mice receiving the heterodimers with adjuvant had approximately 10-fold higher OspA IgG titers one week after the third immunization compared to mice receiving vaccine without adjuvant (Figure 3A). Hence, only vaccine formulations with aluminum hydroxide were further analyzed. In order to study the serotype specific immune response, mice were immunized with 1.0 µg of the individual heterodimers or 3.0 µg of a formulation where the three heterodimers were combined in a 1∶1∶1 ratio (LB-vaccine). Mice receiving 1.0 µg of the corresponding full-length OspA serotype expressed with a lipid moiety were included as controls. The IgG titers were then tested by ELISA, with the respective monomers as coating antigens. The IgG titers for the heterodimers and LB-vaccine were higher or at least comparable to full-length OspA (Figure 3B) and no significant difference was seen between individual heterodimers and the LB-vaccine. This indicates that all six OspA monomers retain the capacity to generate a serotype specific immune response also when linked together and produced as heterodimers. Furthermore, since there was no difference between groups receiving the heterodimers alone or the LB-vaccine, co-formulation of the heterodimers does appear to have neither antagonistic nor synergistic effects. Next, the three heterodimers were tested for protection as individual proteins and when combined in the LB-vaccine (1.0 µg or 3.0 µg, respectively) in different challenge models. Mice were challenged either with ticks harboring *B. afzelii* (OspA serotypes 2) strain IS1 or by s.c. injection of *in vitro* grown *B. burgdorferi* (OspA serotype 1) strain ZS7 or *B. garinii* (OspA serotype 5) strain PHei. Mice immunized with the heterodimer Lip-D1B2B alone or the LB-vaccine were fully protected when challenged with *B. burgdorferi* or ticks harboring *B. afzelii* (Table 6). Similarly, immunizing with heterodimer Lip-D5B6B or the LB-vaccine resulted in significant protection when mice were challenged with *B. garinii* (Table 6). Groups of mice receiving 1.0 µg full-length OspA of the corresponding serotype (Lip-OspA1-His, Lip-OspA2-His or Lip-OspA5-His) were included in each experiment and were fully protected against infection. To further analyze the protection induced by the LB-vaccine, immunizations with decreasing doses (1.5 µg, 0.15 µg and 0.015 µg) were performed and mice were challenged with ticks harboring *B. afzelii* (serotype 2). Significant protection could be observed after immunizing with as little as 0.015 µg, showing that the LB-vaccine is highly efficacious also when administered at very low doses (Table 7).

**Table 6:** The LB-vaccine induces high degree of protection in mice against challenge with spirochetes expressing OspA serotype 1, 2 or 5.

Treatment		Infected/Total		Infected/Total		Infected/Total	
Immunogen	Dose	Exp. 1	Exp. 2	Exp. 3	Exp. 4	Exp. 5	Exp. 6
Lip-OspA1-His	1.0 µg	0/10***	0/10***	–	–	–	–
Lip-OspA2-His	1.0 µg	–	–	0/7***	0/6***	–	–
Lip-OspA5-His	1.0 µg	–	–	–	–	0/10***	0/10***
Lip-D1B2B	1.0 µg	0/10***	0/10***	0/7***	0/3*	–	–
Lip-D5B6B	1.0 µg	–	–	–	–	1/10**	0/10***
LB-vaccine	3.0 µg	0/10***	0/10***	0/5**	0/5**	0/10**	0/10***
Placebo	–	10/10	10/10	6/6	7/7	8/10	10/10

Protection against challenge with *B. burgdorferi* (experiments 1 and 2), *B. afzelii* (experiments 3 and 4) and *B. garinii* (experiments 5 and 6) is shown. *B. afzelii* infected ticks were used for challenge in experiments 3 and 4, and only data from mice where at least one fully fed tick was collected are presented. The *p-*values were calculated with Fisher’s exact test (two tailed); * *p*<0.05, ** *p*<0.01 and *** *p*<0.001.

**Table 7:** The LB-vaccine is highly efficacious when administered at a nanogram dose.

Treatment		Infected/Total	
Immunogen	Dose	Exp. 1	Exp. 2
LB-vaccine	1.5 µg	1/7**	0/9**
	0.15 µg	0/6**	0/10**
	0.015 µg	1/7**	1/9*
Placebo	–	7/7	7/9

Immunized mice were challenged with *B. afzelii* infected ticks, in two separate experiments and only data from mice where at least one fully fed tick was collected are presented. The *p-*values were calculated with Fisher’s exact test (two tailed); * *p*<0.05 and ** *p*<0.01.

### The LB-vaccine induces functional antibodies recognizing all six serotypes of spirochetes

To better understand the potential of the LB-vaccine to induce protective immunity and to evaluate the relationship between the protective immune responses and serological read-outs, immune sera were analyzed by flow cytometry for surface binding and growth inhibition for borreliacidal activity. These assays are of particular interest for OspA serotypes where animal models have not been described and for assessments of the serotype specific immune response in clinical studies. Immune sera from the protection experiments (Table 6) were therefore also analyzed by flow cytometry and growth inhibition. Since binding of antibodies to OspA epitopes presented on the surface of spirochetes is required for elimination in the tick, it can be assumed that flow cytometry may show a better correlation with protection than a total IgG ELISA. Consequently, we analyzed surface binding in more detail. Binding of sera from mice immunized with either the LB-vaccine or the individual heterodimers to the homologous serotype was comparable or even stronger than with sera from mice immunized with the respective full-length OspA, except for serotype 3 (Table 8). The LB-vaccine sera and to some degree also the Lip-D4B3B sera showed a weaker, nonetheless significant, binding to the OspA serotype 3 expressing strain as compared to the full-length OspA induced sera. The low binding activity to OspA serotype 6 by antibodies derived from mice immunized with the full-length OspA, was also analogous to the weak activity seen in the growth inhibition assay (Table 8). The reason for this lower binding activity is not completely understood. However, in the IgG ELISA (Figure 3B) only a slightly weaker immune response was detected, which suggests a lower proportion of functional antibodies induced.

**Table 8:** Functional antibodies induced by the LB-vaccine bind to spirochetes and demonstrate growth inhibiting properties *in vitro*.

	Surface binding			Growth inhibition		
Serotype	FL OspA	Heterodimer	LB-vaccine	FL OspA	Heterodimer	LB-vaccine
1	6693	3244	1231	1250	250	250
2	3641	5311	8934	50	50	50
3	11341	1865	834	n.d.	n.d.	n.d.
4	155	149	225	6250	1250	6250
5	40469	24755	43485	31250	6250	6250
6	57	14211	24634	10	50	250

Surface binding and growth inhibition of sera induced with the LB-vaccine (3.0 µg), individual heterodimers (1.0 µg) or the corresponding full-length (FL) OspA protein (1.0 µg) were analyzed by flow cytometry using spirochetes expressing OspA serotypes 1–6 (serotype 1: *B. burgdorferi* strain ZS7, serotype 2: *B. afzelii* strain PKo, serotype 3: *B. garinii* strain Fr, serotype 4: *B. bavariensis* strain Fin, serotype 5: *B. garinii* strain PHei and serotype 6: *B. garinii* strain KL11). The surface binding data are presented as fluorescence titer, which is the reciprocal of the serum dilution which corresponds to a fluorescence intensity of 100. The growth inhibition data are presented as growth inhibition titer, which is the reciprocal of the lowest serum dilution which gives ≥50% reduction in growth as compared to sera from mice immunized with placebo. Not determined (n.d.).

The ability of the LB-vaccine to induce functional antibodies was further studied by growth inhibition assays. Even though anti-OspA antibody mediated killing of spirochetes in ticks has been shown to occur in a complement independent manner [16], [17], growth inhibition assays can still provide valuable information about the induced immune response. A growth inhibition assay for *B. garinii* serotype 3 could until now not be established, since all strains tested are sensitive to the active guinea pig complement used in the assay, even in the absence of specific antibodies. Analysis of immune sera from the LB-vaccine and full-length OspA immunized mice generated comparable homologous growth inhibition titers (within 1∶5 dilution steps), except for serotype 6 where serum generated with full-length OspA showed an inferior titer (Table 8). The results from both surface staining and growth inhibition correlate for all OspA serotypes, except for OspA serotype 3, in respect to the induction of functional antibodies following immunization with the LB-vaccine.

## Discussion

Earlier OspA based vaccines (LYMErix and ImuLyme) were developed for the North American market and based on full-length OspA from *B. burgdorferi* (serotype 1). However, the presence of additional OspA serotypes in Europe and other LB endemic parts of the world and the limited cross-reactivity due to their heterogeneity, made these vaccines unattractive for regions outside of the US. Indeed, when mice were immunized with full-length OspA from *B. burgdorferi* and subsequently challenged with feral ticks from an LB endemic area in Europe, both *B. afzelii* and *B. garinii* strains could be isolated from the challenged mice [45]. However, mice receiving a combination vaccine with OspA from *B. burgdorferi*, *B. afzelii* and *B. garinii* (serotype 6) were all protected against infection. In another study, protection could be demonstrated when mice were immunized with a monovalent vaccine including full-length OspA from *B. burgdorferi* and subsequently challenged with ticks harboring *B. afzelii* [46]. A more recent approach by Baxter Innovations GmbH towards a vaccine with potentially broad coverage makes use of three chimeric OspA proteins including OspA sequences from six different serotypes [47], [48]. The C-terminal part of each protein is made up of the amino acid sequences from two different serotypes and the N-terminal part is kept intact from one of the two serotypes [47]. Studies in mice have demonstrated vaccine induced protection against *B. burgdorferi* and *B. afzelii* infection as well as the induction of functional antibodies [47], [49]. Data from a clinical phase I trial also showed the induction of antibodies in humans against OspA serotypes 1–6 [48].

The majority of the accessible epitopes of OspA on the surface of spirochetes have been localized to the C-terminal half of the protein [7], [50], [51]. This part of the protein is made accessible to antibodies due to the anchoring to the outer membrane through the N-terminal lipid moiety [6]. Based on these findings, our LB-vaccine approach focuses only on the C-terminal half of OspA from the six clinically most relevant serotypes in Europe and combines them to three heterodimers that are co-formulated in the final LB-vaccine. Our strategy is different from the one by Baxter since we keep the important C-terminal region as far as possible intact for each serotype. The modifications introduced by us are limited to the cysteine mutations, the exchange of the sequence with hypothetical molecular mimicry between a T cell epitope of serotype 1 and hLFA-1 (human leukocyte function-associated antigen-1), the addition of the linker and the lipid moiety. Importantly, we have shown that our heterodimer-approach maintains the native structure of the C-terminal OspA to an extent, which enables the induction of serotype specific and efficacious immune responses and limiting the number of proteins needed in the vaccine formulation. The obtained protection data also indicate that our design maintains the protective epitope characterized for OspA serotype 1 by the monoclonal antibody LA-2 [7]. An immune response to this epitope was shown to correlate with protection in a dog model of infection [18] and it was also shown in the LYMErix phase III clinical trial that the LA-2 equivalent antibody levels were lower in sera from breakthrough cases [52]. By focusing only on the C-terminal part of OspA, our LB-vaccine also possesses a higher molar ratio of potentially protective epitopes compared to a vaccine using the full-length OspA, which might be favorable since a low vaccine dose could still be effective in generating protective immunity.

In a previous study, recombinant OspA fragments including sequences containing a conformational epitope known to provide protection, failed to protect mice from a challenge with spirochetes [53]. Koide and co-workers demonstrated the importance of having the correct structure of the OspA C-terminal domain, in order to keep structural epitopes intact and to provide protection [19]. In line with these findings, we observed superior protection following immunization with any of the five stabilized OspA monomers as compared to the truncated, unmodified OspA from *B. afzelii*. Furthermore, the thermal stability assay as well as the protection studies in mice, showed no significant difference between the five C-terminal variants. Even though the formation of a disulfide bond in M2D-His could not be confirmed with the thermal stability assay, the monomer shows an increased stability compared to the unmodified truncated protein. Therefore, the final selection of stabilization “B” (forming a disulfide bond between the amino acids corresponding to 182 and 269 of OspA serotype 2) and the subsequent translation to additional OspA serotypes was based primarily on expression and purification characteristics as well as efficiency of lipidation. The second cysteine is located in an α-helix at the C-terminal end of the monomer. Since the α-helix is also the attachment site for the linker in the first monomer it is anticipated that extra support at this position is favorable in order to retain the integrity of the heterodimer structure.

The design of the linker was based on two loop-regions from *B. burgdorferi* OspA (strain B31) to introduce flexibility and distance between the monomers, in an effort to reduce steric interference with antibody binding. The protective epitope characterized in OspA serotype 1 by the monoclonal antibody LA-2 is adjacent to the linker attachment site at the C-terminus of the first monomer. Steric restraints at this site do not allow the linker to start with a large residue. Consequently, glycine was chosen as the first amino acid in the linker devoid of a side chain and therefore allowing for high flexibility. The sequence of the linker has been tested in humans before and shown to be safe, since ImuLyme was based on the B31 OspA sequence, including the two relevant loop regions [33], [54].

When the potency of the LB-vaccine was evaluated in mice, significant protection against challenge with spirochetes belonging to OspA serotypes 1, 2 and 5 (representing the three different species *B. burgdorferi*, *B. afzelii* and *B. garinii*, respectively) could be demonstrated. Protection was also confirmed for OspA serotype 2, when mice were challenged via the natural route of infection, namely with ticks. We have so far not been able to identify any *B. garinii* (OspA serotype 3 and 6) or *B. bavariensis* (OspA serotype 4) strains where protection could be demonstrated when immunizing with the homologous full-length OspA. Consequently, protection provided by our LB-vaccine could not be demonstrated for these OspA serotypes. However, to our knowledge data describing protection against these OspA serotypes have not been published. In absence of protection data against OspA serotypes 3, 4 and 6, the protective potency of our LB-vaccine was further supported by immunological assays. The IgG ELISA data showed that the LB-vaccine induced a strong serotype specific antibody response against all six OspA serotypes. The functionality of the antibodies was confirmed *in vitro* as LB-vaccine induced antibodies showed efficient binding to the surface of spirochetes expressing any of the six OspA serotypes and by growth inhibition for five serotypes (except serotype 3) where an assay was successfully set-up. These findings are very encouraging, since these two assays make use of intact spirochetes and it is anticipated that OspA is presented on the surface in a similar manner to the natural situation when present in the tick gut. We believe that our LB-vaccine has a similar transmission blocking action as other OspA based vaccines. OspA induced protection has been shown to rely on binding of anti-OspA antibodies to the spirochetes while residing in the tick gut [9], followed by elimination through a complement independent mechanism [16], [17]. The process takes place before the spirochetes can penetrate the gut epithelium, migrate to the salivary glands and is only effective at the beginning of the tick blood meal [9].

Since protection by OspA based vaccines relies on circulating functional antibodies [9], the generation of high antibody titers is crucial in order to reach long lasting immunity. The lipid moiety is also an important factor influencing the immunogenicity of OspA [33], [55]. When we compared the immunogenicity of the stabilized *B. afzelii* OspA monomer expressed with and without the lipid moiety, we found that the lipid moiety resulted in 10-fold higher antibody titers as determined by ELISA. Further, adsorption of the heterodimers to aluminum hydroxide had a significant effect on their immunogenicity. This together with the finding that as little as 15 ng LB-vaccine protected mice against infection when challenged via the natural route, holds promise for further development including the assessment of vaccine safety and efficacy studies in humans. In summary, our pre-clinical data provides evidence that our rationally designed OspA-based vaccine has the potential for broad protection against Lyme borreliosis.

## References

[1] Centers for Disease Control and Prevention (2013) How many people get Lyme disease? Available: http://www.cdc.gov/lyme/stats/humanCases.html Accessed 2014 May 27.

[2] Hubalek Z (2009) Epidemiology of lyme borreliosis. Curr Probl Dermatol 37: 31–50. 19367096 10.1159/000213069

[3] Lindgren E, Jaenson TG, Organization WH (2006) Lyme borreliosis in Europe: influences of climate and climate change, epidemiology, ecology and adaptation measures. Citeseer.

[4] Rizzoli A, Hauffe H, Carpi G, Vourc HG, Neteler M, et al.. (2011) Lyme borreliosis in Europe. Euro Surveill 16. 21794218

[5] Barbour AG, Tessier SL, Todd WJ (1983) Lyme disease spirochetes and ixodid tick spirochetes share a common surface antigenic determinant defined by a monoclonal antibody. Infect Immun 41: 795–804. 6192088 10.1128/iai.41.2.795-804.1983 PMC264710

[6] Bergstrom S, Bundoc VG, Barbour AG (1989) Molecular analysis of linear plasmid-encoded major surface proteins, OspA and OspB, of the Lyme disease spirochaete Borrelia burgdorferi. Mol Microbiol 3: 479–486. 2761388 10.1111/j.1365-2958.1989.tb00194.x

[7] Ding W, Huang X, Yang X, Dunn JJ, Luft BJ, et al (2000) Structural identification of a key protective B-cell epitope in Lyme disease antigen OspA. J Mol Biol 302: 1153–1164. 11183781 10.1006/jmbi.2000.4119

[8] Schubach WH, Mudri S, Dattwyler RJ, Luft BJ (1991) Mapping antibody-binding domains of the major outer surface membrane protein (OspA) of Borrelia burgdorferi. Infect Immun 59: 1911–1915. 2037351 10.1128/iai.59.6.1911-1915.1991 PMC257942

[9] de Silva AM, Telford SR 3rd, Brunet LR, Barthold SW, Fikrig E (1996) Borrelia burgdorferi OspA is an arthropod-specific transmission-blocking Lyme disease vaccine. J Exp Med 183: 271–275. 8551231 10.1084/jem.183.1.271 PMC2192397

[10] Battisti JM, Bono JL, Rosa PA, Schrumpf ME, Schwan TG, et al (2008) Outer surface protein A protects Lyme disease spirochetes from acquired host immunity in the tick vector. Infect Immun 76: 5228–5237. 18779341 10.1128/IAI.00410-08 PMC2573341

[11] Bunikis J, Garpmo U, Tsao J, Berglund J, Fish D, et al (2004) Sequence typing reveals extensive strain diversity of the Lyme borreliosis agents Borrelia burgdorferi in North America and Borrelia afzelii in Europe. Microbiology 150: 1741–1755. 15184561 10.1099/mic.0.26944-0

[12] Noppa L, Ostberg Y, Lavrinovicha M, Bergstrom S (2001) P13, an integral membrane protein of Borrelia burgdorferi, is C-terminally processed and contains surface-exposed domains. Infect Immun 69: 3323–3334. 11292755 10.1128/IAI.69.5.3323-3334.2001 PMC98291

[13] Fuchs H, Wallich R, Simon MM, Kramer MD (1994) The outer surface protein A of the spirochete Borrelia burgdorferi is a plasmin(ogen) receptor. Proc Natl Acad Sci U S A 91: 12594–12598. 7809084 10.1073/pnas.91.26.12594 PMC45485

[14] Pal U, Li X, Wang T, Montgomery RR, Ramamoorthi N, et al (2004) TROSPA, an Ixodes scapularis receptor for Borrelia burgdorferi. Cell 119: 457–468. 15537536 10.1016/j.cell.2004.10.027

[15] Schwan TG, Piesman J, Golde WT, Dolan MC, Rosa PA (1995) Induction of an outer surface protein on Borrelia burgdorferi during tick feeding. Proc Natl Acad Sci U S A 92: 2909–2913. 7708747 10.1073/pnas.92.7.2909 PMC42328

[16] Gipson CL, de Silva AM (2005) Interactions of OspA monoclonal antibody C3.78 with Borrelia burgdorferi within ticks. Infect Immun 73: 1644–1647. 15731064 10.1128/IAI.73.3.1644-1647.2005 PMC1064931

[17] Rathinavelu S, Broadwater A, de Silva AM (2003) Does host complement kill Borrelia burgdorferi within ticks? Infect Immun 71: 822–829. 12540562 10.1128/IAI.71.2.822-829.2003 PMC145400

[18] Golde WT, Piesman J, Dolan MC, Kramer M, Hauser P, et al (1997) Reactivity with a specific epitope of outer surface protein A predicts protection from infection with the Lyme disease spirochete, Borrelia burgdorferi. Infect Immun 65: 882–889. 9038292 10.1128/iai.65.3.882-889.1997 PMC175064

[19] Koide S, Yang X, Huang X, Dunn JJ, Luft BJ (2005) Structure-based design of a second-generation Lyme disease vaccine based on a C-terminal fragment of Borrelia burgdorferi OspA. J Mol Biol 350: 290–299. 15935380 10.1016/j.jmb.2005.04.066

[20] Fingerle V, Schulte-Spechtel UC, Ruzic-Sabljic E, Leonhard S, Hofmann H, et al (2008) Epidemiological aspects and molecular characterization of Borrelia burgdorferi s.l. from southern Germany with special respect to the new species Borrelia spielmanii sp. nov. Int J Med Microbiol 298: 279–290. 17616434 10.1016/j.ijmm.2007.05.002

[21] Margos G, Vollmer SA, Cornet M, Garnier M, Fingerle V, et al (2009) A new Borrelia species defined by multilocus sequence analysis of housekeeping genes. Appl Environ Microbiol 75: 5410–5416. 19542332 10.1128/AEM.00116-09 PMC2725479

[22] Nadelman RB, Wormser GP (1998) Lyme borreliosis. Lancet 352: 557–565. 9716075 10.1016/S0140-6736(98)01146-5

[23] Ruzic-Sabljic E, Maraspin V, Lotric-Furlan S, Jurca T, Logar M, et al (2002) Characterization of Borrelia burgdorferi sensu lato strains isolated from human material in Slovenia. Wien Klin Wochenschr 114: 544–550. 12422599

[24] Steere AC (2001) Lyme disease. N Engl J Med 345: 115–125. 11450660 10.1056/NEJM200107123450207

[25] Wang G, van Dam AP, Dankert J (1999) Phenotypic and genetic characterization of a novel Borrelia burgdorferi sensu lato isolate from a patient with lyme borreliosis. J Clin Microbiol 37: 3025–3028. 10449497 10.1128/jcm.37.9.3025-3028.1999 PMC85444

[26] Foldvari G, Farkas R, Lakos A (2005) Borrelia spielmanii erythema migrans, Hungary. Emerg Infect Dis 11: 1794–1795. 16422006 10.3201/eid1111.050542 PMC3367353

[27] Maraspin V, Ruzic-Sabljic E, Strle F (2006) Lyme borreliosis and Borrelia spielmanii. Emerg Infect Dis 12: 1177. 16848050 10.3201/eid1207.060077 PMC3291061

[28] Collares-Pereira M, Couceiro S, Franca I, Kurtenbach K, Schafer SM, et al (2004) First isolation of Borrelia lusitaniae from a human patient. J Clin Microbiol 42: 1316–1318. 15004107 10.1128/JCM.42.3.1316-1318.2004 PMC356816

[29] Diza E, Papa A, Vezyri E, Tsounis S, Milonas I, et al (2004) Borrelia valaisiana in cerebrospinal fluid. Emerg Infect Dis 10: 1692–1693. 15503409 10.3201/eid1009.030439 PMC3320289

[30] Strle F, Picken RN, Cheng Y, Cimperman J, Maraspin V, et al (1997) Clinical findings for patients with Lyme borreliosis caused by Borrelia burgdorferi sensu lato with genotypic and phenotypic similarities to strain 25015. Clin Infect Dis 25: 273–280. 9332523 10.1086/514551

[31] Gross DM, Forsthuber T, Tary-Lehmann M, Etling C, Ito K, et al (1998) Identification of LFA-1 as a candidate autoantigen in treatment-resistant Lyme arthritis. Science 281: 703–706. 9685265 10.1126/science.281.5377.703

[32] Steere AC, Drouin EE, Glickstein LJ (2011) Relationship between immunity to Borrelia burgdorferi outer-surface protein A (OspA) and Lyme arthritis. Clin Infect Dis 52 Suppl 3: s259–265. 21217173 10.1093/cid/ciq117 PMC3106239

[33] Erdile LF, Brandt MA, Warakomski DJ, Westrack GJ, Sadziene A, et al (1993) Role of attached lipid in immunogenicity of Borrelia burgdorferi OspA. Infect Immun 61: 81–90. 8418068 10.1128/iai.61.1.81-90.1993 PMC302690

[34] Li H, Dunn JJ, Luft BJ, Lawson CL (1997) Crystal structure of Lyme disease antigen outer surface protein A complexed with an Fab. Proc Natl Acad Sci U S A 94: 3584–3589. 9108020 10.1073/pnas.94.8.3584 PMC20483

[35] Makabe K, Biancalana M, Yan S, Tereshko V, Gawlak G, et al (2008) High-resolution structure of a self-assembly-competent form of a hydrophobic peptide captured in a soluble beta-sheet scaffold. J Mol Biol 378: 459–467. 18367205 10.1016/j.jmb.2008.02.051 PMC2390815

[36] DeLano WL (2002) The PyMOL Molecular Graphics System, DeLano Scientific, Palo Alto, CA, USA. Available: http://www.pymol.org.

[37] Vinayagam A, Pugalenthi G, Rajesh R, Sowdhamini R (2004) DSDBASE: a consortium of native and modelled disulphide bonds in proteins. Nucleic Acids Res 32: D200–202. 14681394 10.1093/nar/gkh026 PMC308760

[38] Pantoliano MW, Petrella EC, Kwasnoski JD, Lobanov VS, Myslik J, et al (2001) High-density miniaturized thermal shift assays as a general strategy for drug discovery. J Biomol Screen 6: 429–440. 11788061 10.1177/108705710100600609

[39] Poljak A, Comstedt P, Hanner M, Schuler W, Meinke A, et al (2012) Identification and characterization of Borrelia antigens as potential vaccine candidates against Lyme borreliosis. Vaccine 30: 4398–4406. 22100635 10.1016/j.vaccine.2011.10.073

[40] Barbour AG (1984) Isolation and cultivation of Lyme disease spirochetes. Yale J Biol Med 57: 521–525. 6393604 PMC2589996

[41] Liang FT, Alvarez AL, Gu Y, Nowling JM, Ramamoorthy R, et al (1999) An immunodominant conserved region within the variable domain of VlsE, the variable surface antigen of Borrelia burgdorferi. J Immunol 163: 5566–5573. 10553085

[42] Schaible UE, Kramer MD, Eichmann K, Modolell M, Museteanu C, et al (1990) Monoclonal antibodies specific for the outer surface protein A (OspA) of Borrelia burgdorferi prevent Lyme borreliosis in severe combined immunodeficiency (scid) mice. Proc Natl Acad Sci U S A 87: 3768–3772. 2339119 10.1073/pnas.87.10.3768 PMC53984

[43] Dani VS, Ramakrishnan C, Varadarajan R (2003) MODIP revisited: re-evaluation and refinement of an automated procedure for modeling of disulfide bonds in proteins. Protein Eng 16: 187–193. 12702798 10.1093/proeng/gzg024

[44] Kiefer F, Arnold K, Kunzli M, Bordoli L, Schwede T (2009) The SWISS-MODEL Repository and associated resources. Nucleic Acids Res 37: D387–392. 18931379 10.1093/nar/gkn750 PMC2686475

[45] Gern L, Hu CM, Voet P, Hauser P, Lobet Y (1997) Immunization with a polyvalent OspA vaccine protects mice against Ixodes ricinus tick bites infected by Borrelia burgdorferi ss, Borrelia garinii and Borrelia afzelii. Vaccine 15: 1551–1557. 9330467 10.1016/s0264-410x(97)00066-2

[46] Fikrig E, Telford SR 3rd, Wallich R, Chen M, Lobet Y, et al (1995) Vaccination against Lyme disease caused by diverse Borrelia burgdorferi. J Exp Med 181: 215–221. 7807004 10.1084/jem.181.1.215 PMC2191810

[47] Livey I, O’Rourke M, Traweger A, Savidis-Dacho H, Crowe BA, et al (2011) A new approach to a Lyme disease vaccine. Clin Infect Dis 52 Suppl 3: s266–270. 21217174 10.1093/cid/ciq118

[48] Wressnigg N, Pollabauer EM, Aichinger G, Portsmouth D, Low-Baselli A, et al (2013) Safety and immunogenicity of a novel multivalent OspA vaccine against Lyme borreliosis in healthy adults: a double-blind, randomised, dose-escalation phase 1/2 trial. Lancet Infect Dis 13: 680–689. 23665341 10.1016/S1473-3099(13)70110-5

[49] Schwendinger MG, O’Rourke M, Traweger A, Savidis-Dacho H, Pilz A, et al (2013) Evaluation of OspA vaccination-induced serological correlates of protection against Lyme borreliosis in a mouse model. PLoS One 8: e79022. 24260146 10.1371/journal.pone.0079022 PMC3832494

[50] Huang X, Yang X, Luft BJ, Koide S (1998) NMR identification of epitopes of Lyme disease antigen OspA to monoclonal antibodies. J Mol Biol 281: 61–67. 9680475 10.1006/jmbi.1998.1930

[51] Sears JE, Fikrig E, Nakagawa TY, Deponte K, Marcantonio N, et al (1991) Molecular mapping of Osp-A mediated immunity against Borrelia burgdorferi, the agent of Lyme disease. J Immunol 147: 1995–2000. 1716290

[52] Steere AC, Sikand VK, Meurice F, Parenti DL, Fikrig E, et al (1998) Vaccination against Lyme disease with recombinant Borrelia burgdorferi outer-surface lipoprotein A with adjuvant. Lyme Disease Vaccine Study Group. N Engl J Med 339: 209–215. 9673298 10.1056/NEJM199807233390401

[53] Bockenstedt LK, Fikrig E, Barthold SW, Kantor FS, Flavell RA (1993) Inability of truncated recombinant Osp A proteins to elicit protective immunity to Borrelia burgdorferi in mice. J Immunol 151: 900–906. 8335917

[54] Sigal LH, Zahradnik JM, Lavin P, Patella SJ, Bryant G, et al (1998) A vaccine consisting of recombinant Borrelia burgdorferi outer-surface protein A to prevent Lyme disease. Recombinant Outer-Surface Protein A Lyme Disease Vaccine Study Consortium. N Engl J Med 339: 216–222. 9673299 10.1056/NEJM199807233390402

[55] Johnson BJ, Sviat SL, Happ CM, Dunn JJ, Frantz JC, et al (1995) Incomplete protection of hamsters vaccinated with unlipidated OspA from Borrelia burgdorferi infection is associated with low levels of antibody to an epitope defined by mAb LA-2. Vaccine 13: 1086–1094. 7491816 10.1016/0264-410x(95)00035-y

